# Prognostic Impact of Frailty in Transcatheter Aortic Valve Implantation

**DOI:** 10.3390/jcdd13030137

**Published:** 2026-03-13

**Authors:** Ivana Jurin, Daniel Unić, Nikola Pavlović, Marin Pavlov, Savica Gjorgjievska, Tomislav Šipić, Šime Manola, Igor Rudež, Ana Šerman, Antonio Bulum, Karlo Gjuras, Irzal Hadžibegović

**Affiliations:** 1 Department of Cardiovascular Diseases, University Hospital Dubrava, 10000 Zagreb, Croatia; nikolap12@yahoo.com (N.P.); marin.pavlov@gmail.com (M.P.); tomislavsipic2@gmail.com (T.Š.); sime.manola@icloud.com (Š.M.); irzalh@gmail.com (I.H.); 2 Department of Cardiac and Transplant Surgery, University Hospital Dubrava, 10000 Zagreb, Croatia; danielunic@gmail.com (D.U.); gjsavica@gmail.com (S.G.); rudi@kbd.hr (I.R.); 3 Department of Nursing, University North, 48000 Koprivnica, Croatia; 4 School of Medicine, Catholic University of Croatia, 10000 Zagreb, Croatia; 5 School of Medicine, University of Zagreb, 10000 Zagreb, Croatia; ana.serman10@gmail.com; 6 Department of Diagnostic and Interventional Radiology, University Hospital Dubrava, 10000 Zagreb, Croatia; antonio.bulum@gmail.com; 7 Department of Family Medicine, Health Centre Bjelovar-Bilogora County, 43000 Bjelovar, Croatia; karlogjuras4@gmail.com; 8 School of Medicine, Josip Juraj Strossmayer University of Osijek, 31000 Osijek, Croatia

**Keywords:** cognitive impairment, Essential Frailty Toolset, frailty, functional status, Katz Index, transcatheter aortic valve implantation

## Abstract

Background: Frailty strongly influences outcomes after transcatheter aortic valve implantation (TAVI), but conventional risk models insufficiently capture functional and cognitive vulnerability. We compared conventional surgical risk scores with multidimensional frailty assessment and a biological score. Methods: This observational registry included 528 consecutive patients with severe symptomatic aortic stenosis undergoing TAVI between January 2019 and November 2024. Frailty was assessed using the Essential Frailty Toolset (EFT), Katz Index, and cognitive screening, alongside French Aortic National CoreValve and Edwards 2 (FRANCE-2) and Age, Creatinine, and Ejection Fraction (ACEF) scores. HALP was calculated as (haemoglobin × albumin × lymphocytes) ÷ platelets. Primary endpoints were 30-day, 6-month, and 1-year all-cause mortality. Secondary outcomes included non-fatal major adverse cardiovascular events (MACE), complications, and quality-of-life improvement. Results: One-year mortality was 12.7%. EFT and Katz Index showed the strongest discrimination for 1-year mortality (AUC 0.72 and 0.75), outperforming EuroSCORE II and STS-PROM (AUC 0.66 and 0.68). After adjustment, EFT (HR 1.91, 95% CI 1.47–2.48), Katz Index (HR 0.57, 95% CI 0.47–0.70, and cognitive impairment (HR 2.24, 95% CI 1.34–3.75) independently predicted 1-year mortality. HALP was not associated with outcomes. FRANCE-2 independently predicted 1-year MACE (HR 1.24, *p* = 0.019). Conclusions: Functional frailty and cognitive impairment add prognostic value beyond conventional comparator models, whereas HALP does not. Brief functional and cognitive screening may help Heart Teams identify patients who need closer peri-procedural optimisation, rehabilitation planning, and discharge support rather than relying on surgical risk scores alone.

## 1. Introduction

Transcatheter aortic valve implantation (TAVI) has transformed the treatment of severe symptomatic aortic stenosis, particularly in elderly and high-risk patients. The PARTNER and CoreValve trials established its safety and efficacy in patients considered inoperable or at high surgical risk [1,2]. Conventional risk stratification has long relied on surgical risk models such as European System for Cardiac Operative Risk Evaluation II (EuroSCORE II) and Society of Thoracic Surgeons Predicted Risk of Mortality (STS-PROM) [3,4]. However, these tools were derived from surgical cohorts and largely calibrated to peri-operative outcomes, which may limit their ability to capture longer-term vulnerability in contemporary TAVI candidates who are typically older, multimorbid, and frequently frail.

Frailty reflects diminished physiological reserve and increased vulnerability to stressors, and it predicts adverse outcomes beyond traditional cardiovascular risk factors [5]. The Essential Frailty Toolset (EFT) has proven robust for predicting mortality and disability after valve replacement, offering a feasible multidimensional bedside assessment [6]. In addition, observational studies have reported that frailty and functional dependence are associated with worse outcomes after TAVI [7,8,9,10]. The European Society of Cardiology and the European Association for Cardio-Thoracic Surgery valvular heart disease guidelines emphasise incorporating frailty and geriatric vulnerability into Heart Team decision-making alongside risk scores and anatomical assessment [11,12]. Beyond functional and cognitive measures, biological vulnerability has attracted interest, including composite laboratory indices. The haemoglobin–albumin–lymphocyte–platelet (HALP) score integrates anaemia, nutritional status, immune competence, and inflammation and has shown prognostic value in other cardiovascular settings [13,14,15].

Despite broad agreement that frailty and geriatric vulnerability matter in TAVI, implementation remains inconsistent and comparative performance of simple bedside tools versus conventional risk models in contemporary, consecutively treated cohorts is still variably reported. Moreover, laboratory-only composite indices such as HALP are increasingly proposed as surrogate markers of ‘biological frailty’, yet their incremental value in TAVI remains uncertain.

Therefore, in a contemporary consecutive TAVI registry, we performed a head-to-head comparison of established surgical risk scores with pragmatic functional and cognitive vulnerability measures (EFT, Katz Index, Clock Drawing Test), and evaluated HALP as a laboratory-based vulnerability index for 30-day, 6-month and 1-year outcomes.

## 2. Materials and Methods

### 2.1. Study Design and Population

This retrospective analysis was performed within the Cardiology Research Dubrava (CaRD) registry (NCT06090591), an institutional observational registry that collects clinical, laboratory, procedural, and longitudinal follow-up data on consecutive patients treated within the TAVI programme. Between January 2019 and November 2024, 528 patients with severe symptomatic aortic stenosis undergoing TAVI were included. Severe aortic stenosis was confirmed by echocardiography, and TAVI indication was adjudicated by a multidisciplinary Heart Team. The study followed the Declaration of Helsinki and was approved by the institutional ethics committee (Approval No. 2025/1204 7).

### 2.2. Baseline Variables

Baseline demographics, comorbidities (diabetes mellitus, chronic kidney disease, chronic obstructive pulmonary disease, coronary artery disease, prior myocardial infarction), echocardiographic variables (including left ventricular ejection fraction and mean aortic gradient), and laboratory measures (haemoglobin, albumin, lymphocyte count, platelet count, creatinine) were recorded.

### 2.3. Risk Scores

Surgical risk was assessed using EuroSCORE II and STS-PROM [3,4]. Frailty was assessed using the EFT according to the original description [6]. Functional dependence was assessed using the Katz Index of Independence in Activities of Daily Living (ADL) [16], which evaluates bathing, dressing, toileting, transferring, continence, and feeding. Cognitive screening was performed using the Clock Drawing Test (CDT), and cognitive impairment was defined as an abnormal CDT [17]. In addition, French Aortic National CoreValve and Edwards 2 (FRANCE-2) and Age, Creatinine, and Ejection Fraction (ACEF) scores were calculated as complementary risk tools [18,19]. All frailty and cognitive assessments (EFT, Katz Index and CDT) were performed pre-procedurally as part of the standard TAVI work-up and were completed usually up to 3 months before the procedure. The HALP score was calculated using the formula: (haemoglobin × albumin × lymphocyte count) ÷ platelet count [13,14,15]. A summary of the components and calculation methods of the ACEF, HALP, Katz Index, EFT, and FRANCE-2 scores is provided in Table 1.

### 2.4. Procedural Details

Valve type (balloon-expandable vs self-expanding), access route, and procedural details were recorded. Complications were defined according to Valvular Academic Research Consortium-2 (VARC-2) criteria, including vascular complications, bleeding, stroke, myocardial infarction, new conduction disturbances requiring pacemaker implantation, paravalvular regurgitation (>2+), acute kidney injury, and intra-procedural mortality [20].

### 2.5. Outcomes

The primary endpoints were all-cause mortality at 30 days, 6 months and 1 year. Secondary outcomes included: (i) a prespecified composite of hard clinical events at 1 year (hard major adverse cardiovascular events; MACEs), defined as non-fatal stroke or transient ischemic attack, non-fatal myocardial infarction, and major bleeding; (ii) an extended post-TAVI adverse events composite capturing additional clinically relevant complications, including hard MACE, complete atrioventricular block, atrial fibrillation, venous thromboembolism, aortic root rupture, endocarditis, hypoattenuated leaflet thickening, and reoperation; (iii) procedural complications defined according to VARC-2 criteria; and (iv) psychiatric complications, such as delirium or acute confusion. Because quality-of-life change was captured only by a single non-validated global follow-up question, it was considered exploratory and was not used to support the main outcome interpretation. These composites were analysed separately because of their heterogeneous mechanisms and prognostic implications. Follow-up was performed at 30 days, 6 months, and 1 year. Mortality status was verified by review of follow-up documentation and cross-checking against available national registry records.

### 2.6. Statistical Analysis

Categorical variables were presented as absolute numbers and percentages. The distribution of continuous variables was assessed using the Shapiro–Wilk test. Variables with a normal distribution were presented as mean ± standard deviation (SD), while variables with a non-normal distribution were presented as median and interquartile range (IQR). Differences between survivors and non-survivors were assessed using the chi-square test for categorical variables, the Student’s *t*-test for normally distributed continuous variables, and the Wilcoxon rank-sum test for non-normally distributed continuous variables.

Cut-off values for each score in predicting one-year mortality were determined using Youden’s J statistic, with the optimal threshold defined at the point of maximum Youden index. For each score, sensitivity, specificity, and area under the receiver operating characteristic curve (AUC ROC) were calculated.

Kaplan–Meier curves were constructed for all risk scores and for cognitive impairment, both for overall survival and for freedom from MACE. For the risk scores, patients were stratified into groups according to the cut-off values derived from Youden’s J statistic. Differences between groups were compared using the log-rank test.

Cox proportional hazards regression models were used to evaluate the association between each risk score and the outcomes of all-cause mortality and non-fatal hard MACE. Results are presented as hazard ratios (HRs) with corresponding 95% confidence intervals (CIs). Model 1 represents the unadjusted analysis. Model 2 is adjusted for the type of transcatheter valve used (balloon-expandable versus self-expandable valves). Model 3 is additionally adjusted for age and sex. Model 4 is further adjusted for body mass index, left ventricular ejection fraction, diabetes mellitus, atrial fibrillation, coronary artery disease, and procedural complications (VARC-2 criteria). Risk scores that inherently include these adjustment parameters in their calculation were excluded from further adjusted analyses. Regression analyses were performed using complete-case data.

A *p*-value < 0.05 was considered statistically significant. Statistical analyses were performed using MedCalc Statistical Software, version 23.2.8 (MedCalc Software Ltd., Ostend, Belgium; 2025).

## 3. Results

Among 528 included patients (Table 2), there were no significant differences in age or sex between survivors and non-survivors at one-year follow-up after TAVI. However, non-survivors more frequently had a history of atherosclerotic disease and prior cardiovascular events. They also presented with lower hemoglobin levels, impaired renal function, and higher preprocedural levels of N-terminal pro–B-type natriuretic peptide and C-reactive protein.

Frailty and functional dependence were common in the overall cohort. A significant difference was observed between survivors and non-survivors in frailty burden, with a median EFT score of 1 versus 2, respectively (*p* < 0.001), and in functional status, with a median Katz Index of 5 versus 4 (*p* < 0.001). Cognitive impairment (abnormal CDT) was also more prevalent among non-survivors (62.7% vs. 35.8%, *p* < 0.001). Frailty measures were not available in all patients: EFT and Katz Index were missing in 120/528 participants (22.7%). This reflected the gradual implementation of routine frailty screening into everyday TAVI work-up during the earlier part of the registry and is acknowledged as an important limitation of the analysis.

**Figure 1:**
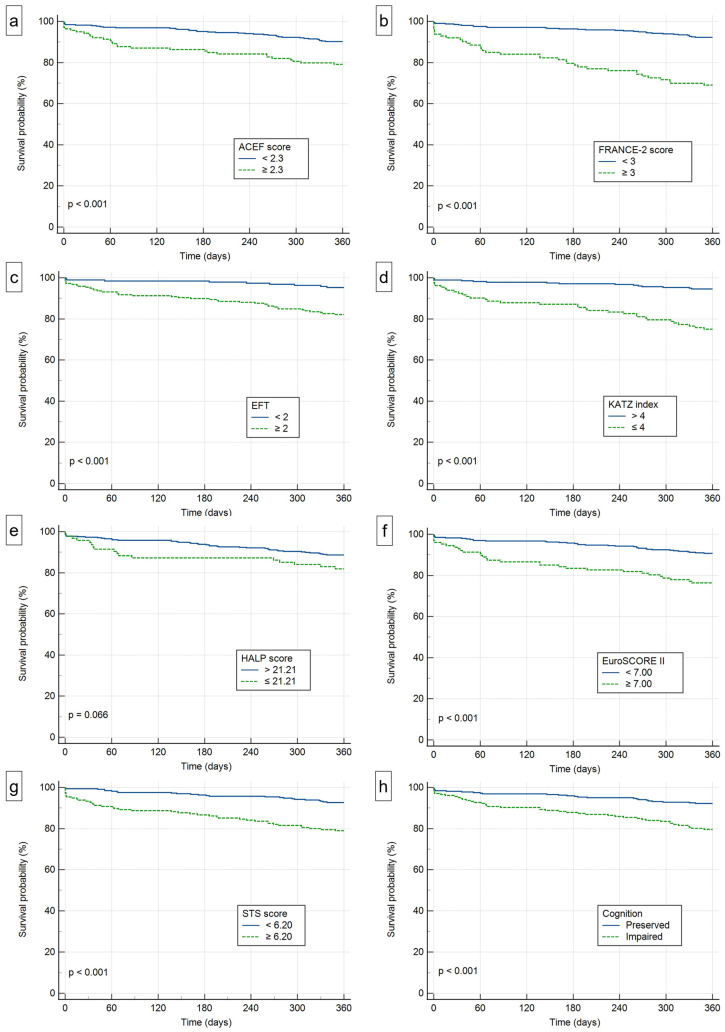
Kaplan–Meier curves for survival probability according to: (**a**) Age, Creatinine, and Ejection Fraction (ACEF) score; (**b**) French Aortic National CoreValve and Edwards 2 (FRANCE-2) score; (**c**) Essential Frailty Toolset (EFT); (**d**) Katz Index of Independence in Activities of Daily Living; (**e**) Hemoglobin, Albumin, Lymphocyte, and Platelet (HALP) score; (**f**) European System for Cardiac Operative Risk Evaluation II (EuroSCORE II); (**g**) Society of Thoracic Surgeons risk (STS) score; (**h**) cognitive impairment status.

All-cause mortality occurred in 2.7% at 30 days, 7.2% at 6 months, and 12.7% at 1 year. Non-fatal hard MACE occurred in 3% by 1 year, while procedural complications as defined by a broad VARC-2-based composite were recorded in 26.1% of procedures (Table 3). Kaplan–Meier analysis (Figure 1) showed progressively reduced survival with increasing frailty and functional dependence (log-rank *p* < 0.001 for both EFT and Katz Index).

Using Youden’s J statistic (Table 4), the optimal EFT cut off for predicting 1 year mortality was 2 (sensitivity 81.3%, specificity 50.3%; AUC 0.720). For Katz Index, the optimal threshold was 4 (sensitivity 68.8%, specificity 72.5%; AUC 0.746).

In unadjusted Cox analyses, both frailty indices were associated with 1-year mortality (EFT: HR 1.91, 95% CI 1.52–2.40, *p* < 0.001; Katz Index: HR 0.56, 95% CI 0.47–0.68, *p* < 0.001). These associations remained significant in the fully adjusted model (EFT: adjusted HR 1.91, 95% CI 1.47–2.48, *p* < 0.001; Katz Index: adjusted HR 0.57, 95% CI 0.47–0.70, *p* < 0.001). Cognitive impairment independently predicted mortality (adjusted HR 2.24, 95% CI 1.37–3.75, *p* = 0.002). HALP was not independently associated with outcomes (HR 1.00, 95% CI 0.99–1.01, *p* = 0.973), while EuroSCORE II, STS PROM, and ACEF demonstrated modest discrimination (AUC 0.60–0.68). For MACE, the FRANC-2 score was the only independent predictor at 1 year after multivariable adjustment (HR 1.24, 95% CI 1.04–1.48, *p* = 0.019). Other risk scores and cognitive impairment were not significantly associated with hard MACE (Table 5).

## 4. Discussion

In this consecutive real-world TAVI registry, multidimensional vulnerability, particularly functional frailty and cognitive impairment, was strongly associated with 1-year outcomes. Bedside functional tools (EFT and Katz Index) and cognitive screening (CDT) were independently associated with 1-year mortality, whereas conventional surgical risk models showed only modest discrimination. These findings align with prior evidence that frailty predicts adverse outcomes after aortic valve replacement [6,7,8,9,10]. The main practical value of the present analysis is that it evaluates brief bedside tools that can be incorporated into routine TAVI work-up and benchmarks them against commonly used risk scores in a contemporary consecutive cohort. Their intended role is complementary: not to replace clinical judgement or anatomical assessment, but to identify vulnerability that is not captured by traditional peri-operative scores.

The discrimination differences observed between frailty tools and EuroSCORE II/STS-PROM should be interpreted cautiously. Numerical performance alone should not drive treatment decisions. Rather, EFT and Katz Index are clinically useful because they capture different but related domains of vulnerability and may prompt specific peri-procedural actions. Patients with higher EFT scores may benefit from pre-procedural optimisation of nutrition, anaemia, mobility, and medication burden, as well as early post-procedural mobilisation and rehabilitation planning. Patients with lower Katz Index scores may require discharge planning focused on activities-of-daily-living support, caregiver involvement, home assistance, or short-term inpatient rehabilitation. When marked multidomain frailty coexists with pronounced ADL dependence and cognitive impairment, the Heart Team should explicitly discuss the possibility of limited functional gain, prolonged dependence, or potential futility despite technically successful TAVI.

Cognitive impairment detected by CDT was common and independently associated with a substantially higher risk of 1-year death. Cognitive vulnerability may influence outcomes through several mechanisms, including reduced participation in rehabilitation, lower adherence to complex therapies, and higher susceptibility to peri-procedural complications such as delirium [21,22]. In practice, an abnormal CDT should trigger closer evaluation of decisional capacity, caregiver support, delirium prevention, medication simplification, and post-discharge supervision rather than serving as a stand-alone reason to deny TAVI. CDT is a feasible screening tool, but it is not a substitute for comprehensive neurocognitive assessment; the observed association should therefore be interpreted as a signal of vulnerability rather than a diagnosis of dementia.

We explored biological vulnerability using HALP, a composite integrating anaemia, nutritional status, immune competence, and inflammation [13,14,15]. HALP did not provide independent prognostic information after adjustment. This negative finding does not negate the relevance of nutrition and body composition in older TAVI recipients since malnutrition and body composition disorders, including osteosarcopenia, have been associated with adverse outcomes after TAVI [23,24]. Rather, our data suggest that a single laboratory composite may have limited incremental value beyond bedside functional and cognitive assessment, and that future work should clarify whether non-linear modelling, recalibrated cut-offs, or combined clinical biomarker approaches can improve risk stratification.

With respect to non-fatal outcomes, we focused on a hard clinical events composite to reduce heterogeneity and improve interpretability. Even so, event rates were relatively low and discrimination was modest, underlining that non-fatal events in this elderly population are influenced by competing risks and multiple pathways. Procedural complications may also contribute to longer-term mortality through bleeding, stroke, acute kidney injury, conduction disturbances requiring pacemaker implantation, prolonged immobility, or loss of functional reserve, even when they are not modelled as baseline predictors. From a clinical perspective, systematic vulnerability assessment may therefore help identify patients at risk of poor recovery and facilitate targeted interventions such as nutritional optimisation, mobilisation, geriatric co-management, delirium prevention, and structured post-discharge planning to reduce potential futility [25].

### Limitations

This observational single-centre analysis is subject to residual confounding despite multivariable adjustment and should be considered hypothesis-generating. Frailty measures (EFT and Katz Index) were missing in 120/528 patients (22.7%); this reflected the gradual implementation of routine frailty assessment into daily TAVI practice during the earlier registry period and complete-case modelling may therefore have introduced selection bias. N-terminal pro-B-type natriuretic peptide was missing in approximately one-third of patients. Cognitive assessment relied on a brief screening test (CDT) rather than a comprehensive neuropsychological evaluation, and performance may be influenced by education, sensory impairment, and motor limitations. EuroSCORE II and STS-PROM were originally developed for peri-operative surgical risk estimation rather than for predicting 1-year outcomes after TAVI, which should be kept in mind when comparing discrimination across tools. We also assessed discrimination for 1-year outcomes using AUC ROC based on 1-year status rather than time-dependent measures. Finally, we did not evaluate patient-reported outcome measures using validated health-related quality-of-life instruments with baseline comparators; therefore, patient-perceived benefit and disability reduction require dedicated prospective assessment.

## 5. Conclusions

In this real-world TAVI cohort, brief bedside assessments of frailty, ADL dependence, and cognitive vulnerability were independently associated with 1-year mortality and provided clinically meaningful information complementary to conventional risk scoring. HALP did not independently predict outcomes. Pragmatic frailty screening appears most useful when it informs specific actions—pre-procedural optimisation, rehabilitation planning, discharge support, caregiver involvement, and more explicit discussion of expected benefit versus possible futility in highly vulnerable patients.

## Figures and Tables

**Table 1:** Overview of the components and calculation methods of the ACEF, HALP, Katz Index, Essential Frailty Toolset (EFT), and FRANCE-2 scores.

Score	Variables Included	Calculation	Risk Interpretation
ACEF [19]	Age; Serum creatinine; Left ventricular ejection fraction (LVEF)	Age (years)/LVEF (%) +1 (if creatinine > 2.0 mg/dL)	Higher score = higher mortality risk
FRANCE-2 [18]	Advanced age; Low BMI; NYHA IV; Pulmonary oedema; Pulmonary hypertension; Critical preoperative state; Respiratory insufficiency; Dialysis; Delivery approach	Sum of predefined clinical risk variables	Higher score = increased early mortality after TAVI
EFT [6]	Chair rise test; Cognitive function; Hemoglobin; Serum albumin	0–5 points total based on predefined thresholds	Higher score = greater frailty and mortality risk
Katz Index [16]	Bathing; Dressing; Toileting; Transferring; Continence; Feeding	1 point per independent activity (0–6)	Lower score = greater functional dependence
HALP [13,14,15]	Hemoglobin; Albumin; Lymphocyte count; Platelet count	(Hemoglobin × albumin × lymphocytes) ÷ platelets	Lower score = worse nutritional/inflammatory status and poorer prognosis

ACEF score: Age, Creatinine, and Ejection Fraction score; EFT: Essential Frailty Toolset; FRANCE-2 score: French Aortic National CoreValve and Edwards 2 score; HALP score: Hemoglobin, Albumin, Lymphocyte, and Platelet score; Katz Index: Katz Index of Independence in Activities of Daily Living.

**Table 2:** Baseline characteristics of the TAVI study population.

Variables	All (*n* = 528) N (%) Mean (±SD) Median (IQR)	Survivors (*n* = 461) N (%) Mean (±SD) Median (IQR)	Non-Survivors (*n* = 67) N (%) Mean (±SD) Median (IQR)	*p*-Value
Demographic and clinical characteristics				
Age (years)	80 (76–83)	80 (76–83)	81 (76–84)	0.308
Sex				0.495
Male	271 (51.3)	234 (50.8)	37 (55.2)	
Female	257 (48.7)	227 (49.2)	30 (44.8)	
Body mass index (kg/m^2^)	28.3 (24.9–31.7)	28.4 (25.0–31.9)	27.8 (24.1–31.0)	0.351
New York Heart Association classification				0.069
Class I	12 (2.3)	10 (2.2)	2 (3.0)	
Class II	244 (46.2)	221 (47.9)	23 (34.3)	
Class III	234 (44.3)	201 (43.6)	33 (49.3)	
Class IV	38 (7.2)	29 (6.3)	9 (13.4)	
Arterial hypertension	462 (87.5)	406 (88.1)	56 (83.6)	0.300
Diabetes mellitus	198 (37.5)	172 (37.3)	26 (38.8)	0.813
Chronic obstructive pulmonary disease or asthma	68 (12.9)	58 (12.6)	10 (14.9)	0.593
Pulmonary hypertension	300 (56.8)	253 (54.9)	47 (70.1)	0.019 *
Atrial fibrillation	216 (40.9)	181 (39.3)	35 (52.2)	0.044 *
Coronary artery disease	212 (40.2)	177 (38.4)	35 (52.2)	0.031 *
Peripheral artery disease	78 (14.8)	62 (13.4)	16 (23.9)	0.025 *
Carotid artery stenosis	90 (17.0)	77 (16.7)	13 (19.4)	0.583
Prior myocardial infarction	86 (16.3)	69 (15.0)	17 (25.4)	0.031 *
Prior cerebrovascular insult	50 (9.5)	37 (8.0)	13 (19.4)	0.003 *
Cognition impaired	207 (39.2)	165 (35.8)	42 (62.7)	<0.001 *
Laboratory parameters				
Hemoglobin (g/L)	127 ± 17	128 ± 17	122 ± 17	0.015 *
Hematocrit (L/L)	0.39 ± 0.05	0.39 ± 0.05	0.37 ± 0.05	0.019 *
Platelet count (×10^9^/L)	207 (167–252)	206 (168–252)	212 (147–241)	0.402
Lymphocyte count (×10^9^/L)	1.4 (1.1–1.8)	1.4 (1.1–1.8)	1.3 (1.0–1.9)	0.609
Total cholesterol (mmol/L)	4.2 (3.4–5.2)	4.2 (3.5–5.2)	4.0 (3.1–5.2)	0.083
Low-density lipoprotein cholesterol (mmol/L)	2.4 (1.8–3.2)	2.4 (1.8–3.2)	2.2 (1.6–3.3)	0.328
High-density lipoprotein cholesterol (mmol/L)	1.3 (1.0–1.5)	1.3 (1.1–1.5)	1.2 (0.9–1.3)	0.001 *
Triglycerides (mmol/L)	1.1 (0.9–1.6)	1.1 (0.9–1.6)	1.1 (0.9–1.4)	0.411
Estimated glomerular filtration rate (mL/min/1.73 m^2^)	57 (42–73)	58 (43–74)	50 (35–67)	0.017 *
C-reactive protein (mg/L)	2.9 (1.3–7.1)	2.8 (1.3–6.9)	4.2 (2.4–9.9)	0.019 *
N-terminal pro–B-type natriuretic peptide (pg/mL)	2315 (781–5610)	2044 (779–5018)	4914 (907–10,191)	0.005 *
Albumin (g/L)	41 (39–44)	41 (39–44)	39 (36–43)	0.002 *
Scores				
ACEF score	1.6 (1.4–2.3)	1.6 (1.4–2.2)	2.0 (1.4–2.9)	0.008 *
FRANCE-2 score	1 (1–2)	1 (1–2)	3 (1–4)	<0.001 *
EFT	2 (1–2)	1 (1–2)	2 (2–4)	<0.001 *
Katz Index	5 (4–6)	5 (4–6)	4 (3–5)	<0.001 *
HALP score	36.10 (24.90–50.60)	36.36 (25.15–50.33)	33.92 (21.04–54.14)	0.572
EuroSCORE II	3.63 (2.22–6.80)	3.45 (2.07–6.28)	5.52 (3.13–11.32)	<0.001*
STS score	4.93 (3.22–8.02)	4.69 (3.09–7.51)	7.43 (4.70–11.97)	<0.001 *
Type of transcatheter valve				0.090
Balloon-expandable valve	337 (63.8)	288 (62.5)	49 (73.1)	
Self-expandable valve	191 (36.2)	173 (37.5)	18 (26.9)	
Access route				0.258
Transfemoral	509 (96.4)	447 (97.0)	62 (92.5)	
Transaxillary	5 (0.9)	4 (0.9)	1 (1.5)	
Subclavian	2 (0.4)	2 (0.4)	0 (0.0)	
Transapical	10 (1.9)	6 (1.3)	4 (6.0)	
Transaortic	2 (0.4)	2 (0.4)	0 (0.0)	
Echocardiographic parameters before TAVI				
Maximum aortic jet velocity (m/s)	4.4 (4.0–4.9)	4.5 (4.0–4.9)	4.3 (4.0–4.8)	0.295
Maximum transvalvular pressure gradient (mmHg)	79 (65–95)	80 (66–95)	74 (60–90)	0.059
Mean transvalvular pressure gradient (mmHg)	47 (38–59)	47 (39–59)	47 (36–58)	0.375
Aortic valve area (cm^2^)	0.7 (0.6–0.9)	0.7 (0.6–0.9)	0.7 (0.5–0.9)	0.817
Left ventricular ejection fraction (%)	56 (45–60)	57 (46–60)	54 (40–60)	0.032 *
Echocardiographic parameters after TAVI				
Maximum aortic jet velocity (m/s)	2.2 (2.0–2.5)	2.2 (2.0–2.5)	2.2 (1.9–2.8)	0.449
Maximum transvalvular pressure gradient (mmHg)	20 (16–25)	20 (16–25)	19 (15–29)	0.820
Mean transvalvular pressure gradient (mmHg)	11 (9–14)	11 (9–14)	12 (9–17)	0.229
Aortic valve area (cm^2^)	1.9 (1.7–2.2)	2.0 (1.7–2.2)	1.9 (1.7–2.0)	0.066
Left ventricular ejection fraction (%)	55 (50–61)	55 (50–61)	52 (41–64)	0.138

ACEF score: Age, Creatinine, and Ejection Fraction score; EFT: Essential Frailty Toolset; EuroSCORE II: European System for Cardiac Operative Risk Evaluation II; FRANCE-2 score: French Aortic National CoreValve and Edwards 2 score; HALP score: Hemoglobin, Albumin, Lymphocyte, and Platelet score; Katz Index: Katz Index of Independence in Activities of Daily Living; STS score: Society of Thoracic Surgeons risk score; TAVI: transcatheter aortic valve implantation. Missing data: albumin (14), body mass index (1), C-reactive protein (2), CCI score (1), EFT (120), HALP score (27), high-density lipoprotein cholesterol (13), Katz Index (120), low-density lipoprotein cholesterol (17), lymphocyte count (13), N-terminal pro–B-type natriuretic peptide (173), STS score (4), total cholesterol (8), triglycerides (10). *: *p* < 0.05.

**Table 3:** Primary and secondary clinical outcomes after TAVI.

Variables	N (%)
Primary outcomes	
Thirty-day all-cause mortality	14 (2.7)
Six-month all-cause mortality	38 (7.2)
One-year all-cause mortality	67 (12.7)
Secondary outcomes	
Hard MACE ^1^	16 (3.0)
Extended MACE ^2^	40 (7.6)
Psychiatric complications	70 (13.3)
Procedural complications ^3^	138 (26.1)

^1^ Hard major adverse cardiovascular event (MACE): non-fatal stroke or transient ischemic attack, non-fatal myocardial infarction, and major bleeding. ^2^ Extended MACE: hard MACE, complete atrioventricular block, atrial fibrillation, venous thromboembolism, aortic root rupture, endocarditis, hypoattenuated leaflet thickening, and reoperation. ^3^ Procedural complications: vascular complications (*n* = 26, 4.9%), bleeding (*n* = 13, 2.5%), stroke (*n* = 8, 1.5%), myocardial infarction (*n* = 2, 0.4%), new conduction disturbances requiring pacemaker implantation (*n* = 49, 9.3%), paravalvular regurgitation (*n* = 31, 5.9%), acute kidney injury (*n* = 4, 0.8%), and intra-procedural mortality (*n* = 5, 0.9%).

**Table 4:** Optimal cut-off values of risk scores for predicting one-year mortality and hard MACE after TAVI determined by Youden’s J statistic.

Score	Cut-Off	Sensitivity	Specificity	Youden’s J	AUC	*p*-Value
One-year mortality						
ACEF score	≥2.3	43.3%	75.9%	0.192	0.601	0.010
FRANCE-2 score	≥3	52.2%	82.9%	0.351	0.699	<0.001
EFT	≥2	81.3%	50.3%	0.315	0.720	<0.001
Katz Index	≤4	68.8%	72.5%	0.413	0.746	<0.001
HALP score	≤21.21	27.0%	82.2%	0.092	0.522	0.601
EuroSCORE II	≥7.00	44.8%	79.6%	0.244	0.664	<0.001
STS score	≥6.20	63.1%	66.5%	0.295	0.680	<0.001
Cognition impaired	Yes	62.7%	64.2%	0.269	0.634	<0.001
One-year hard MACE						
ACEF score	≥1.6	62.5%	50.0%	0.125	0.521	0.782
FRANCE-2 score	≥4	31.3%	86.7%	0.180	0.618	0.083
EFT	≥3	42.9%	76.4%	0.193	0.641	0.028
Katz Index	≤5	78.6%	33.5%	0.121	0.575	0.310
HALP score	≤25.15	43.8%	74.6%	0.184	0.562	0.432
EuroSCORE II	≥2.97	81.3%	38.5%	0.197	0.518	0.784
STS score	≥4.26	87.5%	40.9%	0.284	0.613	0.117
Cognition impaired	Yes	56.3%	61.3%	0.176	0.588	0.176

ACEF score: Age, Creatinine, and Ejection Fraction score; EFT: Essential Frailty Toolset; EuroSCORE II: European System for Cardiac Operative Risk Evaluation II; FRANCE-2 score: French Aortic National CoreValve and Edwards 2 score; HALP score: Hemoglobin, Albumin, Lymphocyte, and Platelet score; Katz Index: Katz Index of Independence in Activities of Daily Living; STS score: Society of Thoracic Surgeons risk score.

**Table 5:** Unadjusted and adjusted Cox regression models of risk scores for predicting one-year mortality and hard MACE after TAVI.

Score	Model 1		Model 2		Model 3		Model 4	
	HR (95% CI, *p*)	C-Index	HR (95% CI, *p*)	C-Index	HR (95% CI, *p*)	C-Index	HR (95% CI, *p*)	C-Index
One-year mortality								
ACEF score	1.36 (1.15–1.61, <0.001)	0.602	1.36 (1.15–1.62, <0.001)	0.612	-	-	-	-
FRANCE-2 score	1.33 (1.23–1.45, <0.001)	0.693	1.33 (1.22–1.44, <0.001)	0.709	-	-	-	-
EFT	1.91 (1.52–2.40, <0.001)	0.710	1.95 (1.54–2.47, <0.001)	0.718	1.97 (1.55–2.50, <0.001)	0.719	1.91 (1.47–2.48, <0.001)	0.761
Katz Index	0.56 (0.47–0.68, <0.001)	0.731	0.55 (0.45–0.67, <0.001)	0.737	0.55 (0.45–0.67, <0.001)	0.744	0.57 (0.47–0.70, <0.001)	0.777
HALP score	1.00 (0.99–1.01, 0.736)	0.522	1.00 (0.99–1.01, 0.758)	0.565	1.00 (0.99–1.01, 0.706)	0.574	1.00 (0.99–1.01, 0.973)	0.708
EuroSCORE II	1.06 (1.03–1.08, <0.001)	0.657	1.06 (1.04–1.08, <0.001)	0.647	-	-	-	-
STS score	1.00 (1.00–1.00, <0.001)	0.674	1.00 (1.00–1.00, <0.001)	0.669	-	-	-	-
Cognition impaired	2.81 (1.71–4.61, <0.001)	0.627	2.90 (1.76–4.76, <0.001)	0.649	2.93 (1.78–4.84, <0.001)	0.659	2.24 (1.34–3.75, 0.002)	0.741
One-year hard MACE								
ACEF score	1.08 (0.69–1.69, 0.726)	0.485	1.08 (0.69–1.70, 0.741)	0.590	-	-	-	-
FRANCE-2 score	1.25 (1.04–1.50, 0.017)	0.634	1.24 (1.04–1.48, 0.019)	0.686	-	-	-	-
EFT	1.53 (1.00–2.34, 0.052)	0.657	1.58 (1.02–2.43, 0.040)	0.685	1.63 (1.05–2.54, 0.031)	0.694	1.42 (0.92–2.21, 0.117)	0.805
Katz Index	0.82 (0.53–1.26, 0.366)	0.582	0.82 (0.52–1.27, 0.366)	0.641	0.80 (0.51–1.25, 0.322)	0.644	0.92 (0.57–1.49, 0.742)	0.790
HALP score	0.99 (0.96–1.01, 0.369)	0.566	0.99 (0.96–1.02, 0.391)	0.623	0.99 (0.96–1.01, 0.355)	0.641	0.99 (0.96–1.02, 0.492)	0.776
EuroSCORE II	0.98 (0.88–1.09, 0.678)	0.470	0.98 (0.88–1.09, 0.692)	0.558	-	-	-	-
STS score	1.06 (0.99–1.12, 0.082)	0.623	1.05 (0.99–1.02, 0.110)	0.658	-	-	-	-
Cognition impaired	2.16 (0.81–5.81, 0.126)	0.597	2.29 (0.85–6.15, 0.101)	0.647	2.43 (0.89–6.66, 0.084)	0.674	1.98 (0.69–5.64, 0.150)	0.777

ACEF score: Age, Creatinine, and Ejection Fraction score; EFT: Essential Frailty Toolset; EuroSCORE II: European System for Cardiac Operative Risk Evaluation II; FRANCE-2 score: French Aortic National CoreValve and Edwards 2 score; HALP score: Hemoglobin, Albumin, Lymphocyte, and Platelet score; Katz Index: Katz Index of Independence in Activities of Daily Living; STS score: Society of Thoracic Surgeons risk score. Model 1: unadjusted; Model 2: adjusted for valve type (balloon-expandable versus self-expandable valves); Model 3: additionally adjusted for age and sex; Model 4: further adjusted for body mass index, left ventricular ejection fraction, diabetes mellitus, atrial fibrillation, coronary artery disease, and procedural complications (Valvular Academic Research Consortium-2 criteria). Complete-case data were used for regression analyses.

## Data Availability

The data presented in this study is available on request from the corresponding author.

## Abbreviations

The following abbreviations are used in this manuscript:

ACEF	Age, Creatinine, and Ejection Fraction
ADL	Activities of Daily Living
AUC ROC	Area under receiver operating characteristic curve
CDT	Clock Drawing Test
CI	Confidence interval
EFT	Essential Frailty Toolset
EuroSCORE II	European System for Cardiac Operative Risk Evaluation II
FRANCE-2	French Aortic National CoreValve and Edwards 2
HALP	Haemoglobin–albumin–lymphocyte–platelet
HR	Hazard ratios
IQR	Interquartile range
MACE	Major adverse cardiovascular event
PROM	Patient-reported outcome measure
SD	Standard deviation
STS-PROM	Society of Thoracic Surgeons Predicted Risk of Mortality
TAVI	Transcatheter aortic valve implantation
VARC-2	Valvular Academic Research Consortium-2
